# Microneedle-Based Glucose Sensor Platform: From *Vitro* to Wearable Point-of-Care Testing Systems

**DOI:** 10.3390/bios12080606

**Published:** 2022-08-06

**Authors:** Jian Ju, Lin Li, Sagar Regmi, Xinyu Zhang, Shixing Tang

**Affiliations:** 1 Wenzhou Institute, University of Chinese Academy of Sciences, Wenzhou 325001, China; 2 Oujiang Lab, Wenzhou 325001, China; 3 School of Ophthalmology & Optometry, Wenzhou Medical University, Wenzhou 325035, China; 4 Department of Pharmacology, School of Medicine, Case Western Reserve University, Cleveland, OH 44106, USA; 5 Department of Epidemiology, School of Public Health, Southern Medical University, Guangzhou 510515, China

**Keywords:** microneedle, materials, continuous glucose monitoring, wearable biosensor, electrochemical, surface-enhanced Raman spectroscopy

## Abstract

Significant advanced have recently been made in exploiting microneedle-based (MN-based) diabetes devices for minimally invasive wearable biosensors and for continuous glucose monitoring. Within this emerging class of skin-worn MN-based sensors, the ISF can be utilized as a rich biomarker source to diagnose diabetes. While initial work of MN devices focused on ISF extraction, the recent research trend has been oriented toward developing in vivo glucose sensors coupled with optical or electrochemical (EC) instrumentation. This outlook highlights the essential characteristics of the sensing mechanisms, rational design, sensing properties, and applications. Finally, we describe the opinions about the challenge and prospects of optical and EC MN-based device platforms for the fabrication of wearable biosensors and their application potential in the future.

## 1. Introduction

Diabetes mellitus is increasingly recognized as a serious worldwide public health concern; looking back at the growth rate of patients over the past 40 years, it can be expected to double by 2030 compared to that in 2015 [1,2]. One of the main roadblocks to disease control requires sensitive and reliable point-of-care testing (POCT) technology and should be able to provide quantitative results for disease diagnostics and monitoring [3]. Tight glycemic control is widely accepted for the diagnosis and monitoring of diabetes. Up to now, many types of techniques have been developed to measure blood glucose in all stages of treatment and disease management of diabetic patients [4,5,6]. Most of the above methods require the frequent withdrawal of fresh blood from the fingertip, which creates pain and is inconvenient with the potential risk for cross-contamination. Moreover, it cannot keep up with the demand for the real-time monitoring of blood glucose where the frequency of puncture is necessary daily, and the physiological signals which are missed may delay the medical treatment in time. Wearable sensors can alert the user regarding health abnormalities and are actively being developed by scientific researchers and medical diagnostics companies to achieve continuous monitoring of diseases [7]. Blood [8], sweat [9], saliva [10], and interstitial fluid (ISF) [11] are common detection samples in the design of wearable biosensors. In comparison with the other peripheral biofluids, the ISF contains abundant physiological information and exhibits a close correlation with blood samples due to the transcapillary exchange between blood and cells. Thus, the ISF can be utilized as a biomarker source to design the wearable biosensor to diagnose diabetes [12].

In the past few decades, microneedle (MN) patches have attracted extensive attention for their unique advantages for the collection of ISF samples [13]. The MN patches in micron-scale sizes of less than 1500 μm in length are normally arranged in an array form with up to hundreds of pieces [14]. In comparison with the hypodermic needles, the penetration by MN through the superficial skin usually does not reach the nerves and blood vessels in the deeper layers of the dermis, thus enabling painless and blood-free ISF collection devices [15]. Such convenient and efficient extraction ISF process promotes the development of a variety of biosensors, such as blood glucose [16], electrolyte [17], pH [18], drug metabolism [19], etc., [20]. When the MN device is indwelled under the skin, it opens a test window on the skin after being combined with optical, electrical, and other detection technologies to achieve real-time monitoring of the biological target components in the ISF [20]. Since non-invasive biosensing systems remain an elusive goal to date, the MN may be an ideal device to fabricate minimally invasive wearable biosensors at this stage of development. Innovative fabrication techniques MNs have been developed so far with various materials such as silicon, glass, ceramic, metal, polymers, carbohydrate etc., [21]. To realize particular functional requirements, people have also prepared microneedles of different shapes such as solid MN, hollow MN, dissolving MN, swellable MN, and coated MN [22]. Nevertheless, the research of wearable glucose biosensors faces considerable challenges. On the one hand, the strong mechanical strength, biocompatibility, complex manufacturing, and difficult integration with the test technologies are perceived as the four main obstacles to the development of MN wearable glucose sensors. On the other hand, the skin is sensitive, and it is easy to cause skin eruption by allergic reactions when the MN indwelling in the skin, the leaking of tissue fluid, and sweating cause interference with the detection sensitivity of wearable sensors. Therefore, there is still an urgent need that the MN-based wearable biosensor to be worn on the skin for a long time without discomfort and would provide timely feedback to patients’ diagnosed information.

Over the past few years, several reviews focusing on MN-based biosensor devices emphasized various aspects of NM biosensors, including the manufacturing method, materials science, glucose monitoring, and different sensing formats. However, a comprehensive review that focuses on MN devices with the great potential to prepare a miniaturized wearable device for continuous monitoring of glucose remains to be presented. In this review, we summarize the current advances in the development of skin-worn and minimally invasive MN-based sensor systems. Firstly, we detail the employed strategies and materials for the preparation of minimally invasive epidermal MN. Secondly, we particularize the different test technologies for monitoring glucose levels, such as the colorimetric, electrochemical, and Raman spectrum. Then, we present the potential of MN-based devices for individual long-time monitoring and routes to incorporate them onto one wearable sensor platform. Finally, we describe the opinions about the challenge and outlook of MN-based device platforms for the fabrication of wearable biosensors and their ability to open new avenues in the field of wearable biosensors.

## 2. Overview of Fabrication of MN 

From the start of work in 1976, many strategies have been developed for the preparation of MN, such as lithography, molds, etching [23], and three-dimensional printing [24]. The MNs can be classified into different groups based on materials, shape, and application orientation. The different materials that were selected so far for the preparation of the MN are silicon, glass, ceramic, metal, polymers, and carbohydrates. Based on their working principle, MNs are currently manufactured as solid, hollow, dissolvable, and swellable. 

### 2.1. Materials for MN Fabrication

#### 2.1.1. Metals

**Figure 1:**
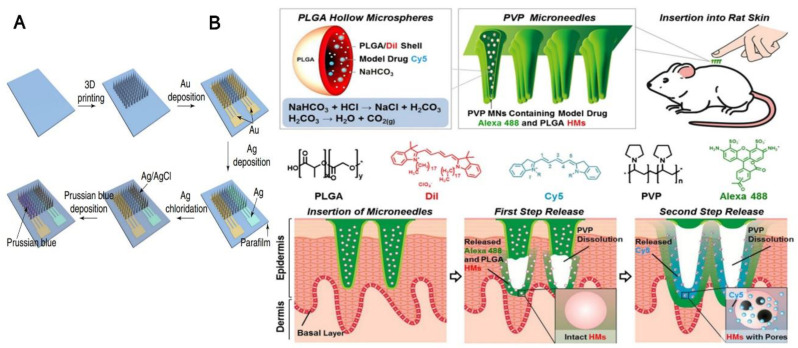
(**A**) General diagram showing the overview of the process of preparing biosensors where 3D printing was used to fabricate the MN arrays using the technique of gold deposition followed by silver deposition and chlorination along with electroplating process to obtain a Prussian blue layer, “Reprinted with permission from ref. [26]. 2021, Nature”; (**B**) The upper panel shows the design of PVP microneedles using PLGA hollow microspheres and illustration of insertion into rat skin. The Alexa 488 and PLGA HMs is shown in first step release followed by cy5 in second step release with PVP dissolution. “Reprinted with permission from Ref. [36]. 2012, Elsevier”.

Metal is the most common material for the preparation of the MN due to its ease of fabrication. Among them, stainless steel and titanium have attracted much attention because of their good mechanical strength and controllable structure [25]. Chen’s lab has developed a simple, rapid, and inexpensive strategy to fabricate hollow metal MN arrays using chemically etched silica needles as templates. In addition to that, some noble metals such as gold and silver are usually applied in MN to improve the performance of the sensor or drug delivery, as shown in Figure 1A [26]. Liu’s group reported a strategy for preparing the stainless-steel MN (Admin Patch 1200, AdminMed, Sunnyvale, CA, USA), which is coated with silver (Ag) to detect analytes at a depth of more than 700 mm below the surface of a skin phantom [27]. The Ag layer in the Ag-coated stainless-steel MN is found to be effectively enhancing the Raman signal. However, the wide use of these metal and stainless-steel needles would yield sharp waste and the potential biohazardous of used MN without adequate sterilization [28].

#### 2.1.2. Silicon

Silicon is a very important material for preparing non-metallic MN due to its excellent mechanical strength and shape plasticity [23,29]. Different lengths and shapes of silicon-based MN are reported by deep reactive ion etching and photolithography methods [30]. Yan and Gale used a deep reactive ion etching technique to fabricate solid silicon MN arrays where the needle densities ranged from 400 to 11,900 needles/cm [31,32]. Such silicon-based MN exhibited better performance for diagnosis and drug delivery. However, some part of silicon-based MN is easy to break under the skin, which causes potential biological risks [33]. Moreover, the expensive instrument to prepare MN is a key factor that limits its wide application [34].

#### 2.1.3. Polymer

Some very promising technology is focused on polymers MN since they are biocompatible, biodegradable, mechanically strong for skin insertion, and easy to manufacture. The most common method for producing polymer MN is the reverse mold method, as shown in Figure 1B. A wide variety of polymers including poly (methyl methacrylate) (PMMA) [35], poly (lactic-co-glycolic acid) (PLGA) [36], polyglycolic acid (PGA) [37], cyclic-olefin copolymer, poly (vinylpyrrolidone) (PVP) [38], poly (vinyl alcohol) (PVA) [39], methacrylate hyaluronic acid (MHA) [15] and SU-8 [40] are reported for MN preparation. Different from metals and silicon-based materials, polymers can be prepared by dissolving and swellable MN, which has unique advantages in the application of transdermal delivery and body fluid extraction. 

Dissolving polymer MN have a unique application in the drug delivery field, which can dissolve within minutes or days and completely reabsorb in the skin, resulting in no biohazardous sharps [41]. Dissolving polymer MN gives a new way to improve the drug or biomolecular loading rate and accurately control the loading dose in comparison with the solid MN [37,42,43]. The preparation process of dissolving polymer MN is relatively simple and needs a two-stage mild fabrication process; the polymer is filled into the MN mold, then using a long centrifugation time to overcome the constraints of surface tension and solution viscosity [44,45,46]. It is much safer compared with metals and silicon MNs.

The swellable MN has gained significant interest because they swell by absorbing ISF under the skin, and the MN body is maintained without dissolving [47,48]. Such swelling behavior of the MN provides a new strategy for drug delivery and body fluid extraction [49]. Karp’s group first discusses the change of shape, such as swellable tip interlocking with tissue. In this process, the cone-shaped needles can be inserted into the tissue in a dry (stiff) state; once the needle tips are in contact with the water in the tissue, the swollen needle tip localized in the tissue [50]. Xu’s lab developed a swellable MN platform based on meth-acrylated hyaluronic acid (MHA) crosslinked by ultraviolet (UV) exposure that can rapidly and efficiently extract ISF, which greatly facilitates the subsequent offline timely analysis of metabolites [15]. In comparison with the dissolving polymer, swellable MNs exhibited strong potential application in the precise extraction of body fluid field.

#### 2.1.4. Temperature-Dependent Materials

Carbohydrate-based materials are typical temperature-dependent materials, and they can be employed to make MN by micro-molding in high processing temperatures and drawing lithography, including the chitosan [51], sugar [52], galactose [53], polysaccharide [54], and galactose [55] which are widely used in the fabrication of MN. The carbohydrate-based MNs usually have a fundamental part in wellness management, which provides stimulating revolutions in drug delivery [22,56]. It must be pointed out that carbohydrates with an approval history in US Food and Drug Administration (US FDA) and classified as “generally recognized as safe” (GRAS) [57,58]. Xu’s lab first prepared a cry-MN (cryonics) patch. The patch was fabricated by step-by-step cryogenic in predesigned MN molds containing pre-suspended cells. The cryonic MNs can easily pierce the skin and deliver loaded living cells into the skin. This work provides new ideas for improving MN technology to intervene in the higher-level precision of the medical field.

### 2.2. Shape of MN

#### 2.2.1. Solid

Solid is the most common shape of MN, and it is easy to prepare and usually has strong mechanical strength [59]. Solid MNs are usually used to create micro holes in the skin. The solid MNs are mostly applied to create microchannels in porcine. Different from other needles, the MN tip depth of solid MN does not reach the pain receptor. Thus, this process could be painless and improve patient compliance [60]. The microchannels created by the solid MN provide a simple way to diffuse drugs and extract ISF. The solid MN are regarded as devices so far for cosmetology, vaccination, drug delivery, etc., [33,46,61,62,63]. However, the solid MN bears limitations for drug delivery and ISF extraction due to the low drug loading rate and low efficiency of extracting body fluids. Therefore, hollow and coating MN has been successively developed to solve the above problems.

#### 2.2.2. Coated MN

The coated MNs are based on solid MNs, macromolecules [64], small molecules, vaccines [65], or micron-size particles [53] that have close contact with the body of the needle [66]. In order to successfully create coatings on MN, different coating methods have been reported, such as dip coating [66], immersion coating [67], inkjet Printing [68], drop coating [69], and layer-by-layer coating [70]. Among them, the dip-coating method is the most utilized, but it has the problem of inherently suffering from poor drug delivery efficiencies. Therefore, if the dip-coating approach is used for layer-by-layer coatings, higher drug delivery efficiencies could be achieved [71,72]. More importantly, such coated MN gives the MN new functions, such as significantly improving its optical performance and expanding the application of the microneedle in the field of biosensing [28]. Van Duyne’s Lab used the active gold nanorods coated on the MN array surface to design a pH-sensitive biosensor; this sensor can quantitate pH over a range of 5 to 9 and can detect pH levels in an agar gel skin phantom and human skin in situ [73]. 

#### 2.2.3. Hollow MN

The hollow MN has been developed because it allows aqueous drugs to flow through the lumen and into the skin, leading to faster rates of drug delivery than solid MN, which relies on the diffusion of drugs in the skin [74,75]. It must be noted that hollow needle fabrication is significantly difficult, and those possessing a high aspect ratio lack the internal support structure common to solid needles [76]. In addition, the less mechanical strength and non-uniform insertion of the hollow MN may cause transverse bending and limit their potential for drug delivery and extracting biological fluids through the skin. However, embedding open channels on hollow MN is a novel approach, providing a passage through the skin for light, which provides a new idea for collecting light signals under the skin. Liu’s lab proposed a hollow agarose MN with the pipette tip as the hollow mold and adopted Tollen’s method to form the thin silver layer as the SERS-active film hollow MN-SERS device, expanding the application of microneedles in the field of biosensing [28].

## 3. Microneedle Used for Glucose Monitoring

In the early stages, MN has mainly been used for the extraction of ISF or blood, on which the glucose level was measured by offline analysis technology. ISF can readily be extracted from the micro-hole created by MN; the concentration of glucose in the ISF is similar to it in the blood sample due to the energy transfer between blood and ISF [77,78]. Despite the good correlation, glucose monitoring in ISF is challenging due to its diffusion gap from capillaries to skin [79]. This provides a new way for the future development of wearable biosensors that can be worn for a long time. 

### 3.1. Colorimetric-MN Glucose Sensor

E.V. Mukerjee et al. reported the fabrication of a silicon MN array used for the ISF extraction from the skin in 2004, each needle of the 20 × 20 MN array has a 200–350 μm tall needle shaft along with a base diameter of 120 μm on 300 μm centers. However, there exists an initial 20–30 min latent time that is required to generate ISF that is enough to fill the bore hole of the MN. Then, then the color change from clear to deep blue shows that there is around 80 to 120 mg/dL in the extracted fluid [80]. In 2017, a novel MN sensing concept was developed by Xu et al. where they designed and fabricated a novel swellable MN platform based on the meth acrylated hyaluronic acid (MeHA); the obtained MN can rapidly and efficiently extract ISF from the skin, extract ≈1.4 mg ISF within 1 min. The extracted skin ISF can be recovered from the MN patch by centrifugation for the subsequent offline colorimetric glucose analysis. This proof-of-concept research opens a new avenue for the progress of MN-based microdevices, which has the potential application of sampling ISF as well as detection of minimally invasive metabolites. Kim et al. designed and fabricated a platform with porous MN on a paper substrate (PMP) for the quick analysis of absorbed samples. The paper-based sensors, as well as porous MNs, have a perpendicular through-whole fluidic channel: where the absorbed sample via MN flows to the sensor. The MN, which is biodegradable, has interconnected pores of 5–10 μm in diameter. The colorimetric method is used to indicate the glucose concentration by the TMB dye oxidization, and blue color development occurs in the presence of hydrogen peroxide [81].

The Gu’ lab designed a glucose-responsive colloidal crystal (GCC) patch for minimally invasive, uncomplicated as well as naked-eye recognizable glucose colorimetric monitoring. To realize the application of this material in situ detection of glucose, they developed a secondary modification strategy that integrated the GCC with MN. Such core-shell MN structure by the assembly of the soft GCC on hard MN can be used to maintain the stimulus-responsive property of the GCC as well as support sufficient mechanical strength of MN to prick the skin. The skin showed very less inflammation after GCC-MN insertion in comparison to the untreated skin, which clearly proves the biosafety of the GCC-MN patch [16].

Xu and Li et al. reported an ingenious colorimetric dermal tattoo biosensor, which was manufactured by an MN patch for parallelly detecting the multiple health-related biomarkers ex vivo as well as in vivo. Then these MN patches transfer the reagents of colorimetric reaction into the dermis with very less invasion, which can form dermal tattoo biosensors. This biosensor reveals a change in color with respect to differences in pH, glucose, uric acid, as well as the temperature of the body. These alterations can be read by naked eyes for qualitative detection as well as captured using the camera of a smartphone. Such kind of colorimetric dermal tattoo biosensor is very helpful not only in health management but also in monitoring the disease. 

### 3.2. Electrochemical-MN Glucose Sensors

The electrochemical-MN (EC-MN) glucose biosensor perfectly combines the MN with electrochemical detection technologies, which produces a series of glucose biosensors with excellent performance. The EC sensor is widely used for the development of glucose biosensors, comprising enzymatic and non-enzymatic sensors as shown in Table 1. The concept of enzyme-based glucose electrochemical sensors is dependent to monitors the oxygen consumption according to the enzyme-catalyzed reaction. However, the non-enzymatic EC glucose sensors are depended on the direct electrochemical oxidation of glucose [82]. The combination of MN patch technology paves another avenue for the development of a novel device for the in vitro and in vivo measurement of glucose in the ISF. 

#### 3.2.1. Enzyme-Based EC Glucose Sensor

**Figure 2:**
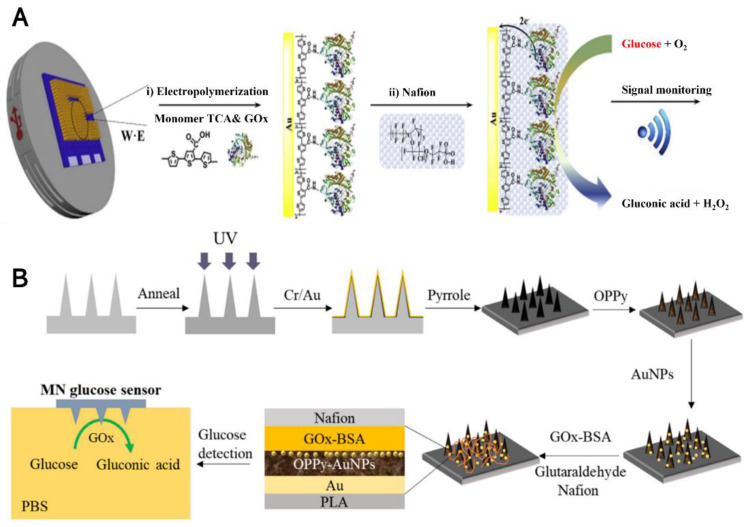
(**A**) Schematic representation of the reaction mechanism for pTCA was employed to stable immobilize GOx onto the electrode surface, “Reprinted with permission from Ref. [92]. 2019, Elsevier”; (**B**) The fabrication process of Nafion/GOx/AuNPs/OPPy/AuMNs array EC glucose sensor, layer by layer structure, and the glucose monitoring scheme in PBS, “reprinted with permission from Ref. [93]. 2020, Elsevier”.

Wang’s lab described a novel minimally invasive bicomponent MN sensing device for the electrochemical monitoring of the excitatory neurotransmitter glutamate and glucose in their earlier research in 2011 [83]. The solid and porous needles were prepared, respectively, and a hollow MN cover was then placed over the solid MN substrate; the enzymes glutamate and glucose oxidase were modified on the solid needle surface using a poly(o-phenylenediamine) thin-film. The electrochemical signal response is well defined and observed over the pathophysiological range (0–14 mM) in a buffer matrix. The Aderson’s group reported using stainless steel (316L) 2D MN array as amperometry glucose-senor smart patch. They utilized a conduction polymer ploy(3,4-ethylenedioxythiophene) as an immobilization agent for the glucose oxidase (GOx). After platinum-coated on the needles, the sensor exhibited linearity between 36 and 432 mg dL^–1^ glucose (2–24 × 10^−3^ M, S/N = 10.7). The Antiochia lab reported a highly porous gold MN based on a second-generation biosensor for minimally invasive monitoring of glucose in an artificial interstitial fluid. The gold surfaces of MNs, which are highly porous, were first modified by immobilization of 6-(ferrocenyl)hexanethiol (FcSH). This acts as a redox mediator. Then, it was again immobilization by a flavin adenine dinucleotide glucose dehydrogenase (FAD-GDH) enzyme. The MN-based FcSH/FAD-GDH biosensor exhibited an extended linear range (0.1–10 mM), high sensitivity (50.86 µA cm^−2^ mM^−1^), and stability for the test glucose in the ISF sample [91]. Along with the development of nanotechnology, combining nanomaterials with MN technology promotes the electrochemical MN glucose sensor to high performance and biological application; the preparation strategy is shown in Figure 2A. Guo’s lab developed a polylactic acid (PLA) based MN glucose sensor [93], as shown in Figure 2B. The gold film was used as the conductive layer; through modification by overoxidized polypyrrole (OPPy), gold nanoparticles (AuNPs), glucose oxidase (GOx), and Nafion membrane the glucose MN sensor was fabricated successfully. In this process, OPPy provides a good place for AuNPs electrodeposition and GOx immobilization. This novel glucose sensor showed a linear range from 0 to 2.6 mM with a sensitivity of 8.09 μA/mM and a limit of detection (LOD) of 40 μM in phosphate buffer solution (PBS). With ever more sophisticated enzyme-based electrochemical glucose biosensors development, was spawned an in vivo application of “Continuous Glucose Monitoring”. The Cui group reported a biosensing device named integrated MN, which was inserted into the skin of a mouse. Interestingly, it showed accurate sensing performance for monitoring the subcutaneous glucose levels in normal as well as diabetic mice. The device was manufactured with 3D printing technology; then, gold evaporation was performed to obtain the working electrode and an adhesion layer of the reference electrode. Then, the blue Prussian layer was used to immobilize the glucose oxidase (GOD) on the working electrode. The MN biosensing device exhibited a linear detection range on the scale of 0.8 to 24 mM with a sensitivity of 0.0741 ± 0.0004 μA/mM in phosphate-buffered saline (PBS). More interestingly, this MN biosensing device used in monitoring glucose in normal and diabetic mice for 7 days shows that the interstitial level of glucose has a strong correlation with the level of blood glucose with a value of high correlation coefficient (0.9032) for the normal mouse as well as (0.9239) for the diabetic mouse [26]. 

Voelcker’ lab first reported high-density silicon MNs (≈9500 MN cm^−2^), which are used to make a three-electrode patch for monitoring the level of glucose. The surface of the Si MN array was first coated using a thin layer of gold (Au-Si-MNA) and then modified to conjugate dendrimers, which has a redox mediator (ferrocene cored poly(amidoamine) dendrimers [Fc-PAMAM]) followed by the catalytic bioreceptor glucose oxidase (GOx) as work electrode. The main features of the MNA glucose patch in ISF showed very good selectivity (0.1622 µA mm^−1^ cm^−2^) with a working concentration on the scale of 1–9 mM glucose, and it has a detection limit (0.66 mM) (and a correlation coefficient (0.9995). In the case of in vivo glucose monitoring experiment, the working counter and reference electrode were pressed by a finger onto the back skin of a mouse, and the test result demonstrated that this novel glucose sensor patch has the capability to observe the alteration in the level of ISF glucose [95].

#### 3.2.2. Non-Enzyme Electrochemical-MN Glucose Sensor

**Figure 3:**
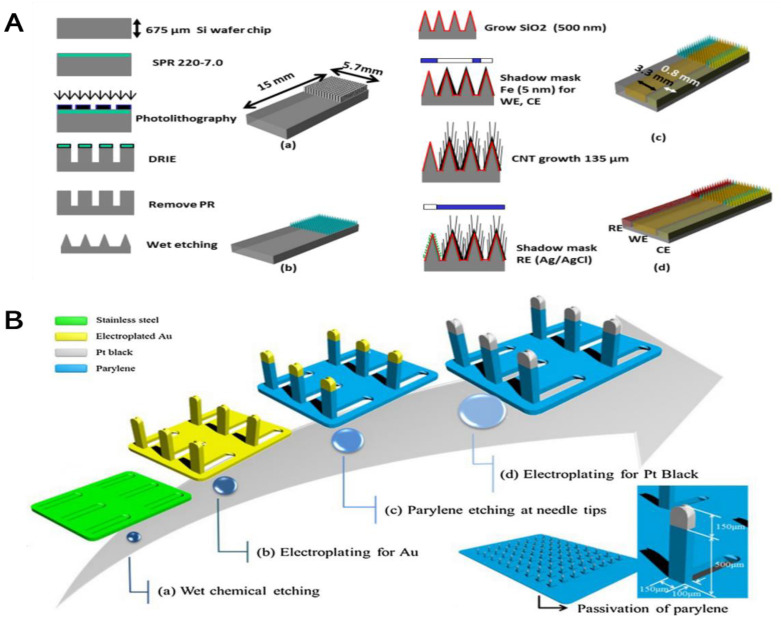
(**A**) Illustration showing non-enzymatic EC MN-based glucose sensor (a) the deep reactive ion etching of silicon was performed to make a rectangular shape of pillar array; (b) the wet etching of the rectangular pillar Si array was performed to generate a sharp Si needle array; (c) the iron deposition technique was performed using a shadow mask as well as MWCNT growth followed by platinum based nano-particles electroplating; and (d) The silver deposition was performed using a shadow mask as well as it can form silver/silver chloride electrode as a reference, “reprinted with permission from Ref. [96]. 2013, MDPI”; (**B**) The fabrication sequences of three-dimension MN array was shown using the platinum black catalytic layer for the patch kind of non-enzymatic detection of level of glucose “reprinted with permission from Ref. [97]. 2016, Elsevier”.

In clinical diagnosis, enzyme biosensors expose some deficiencies, such as the enzymes must be available to catalyze a specific biochemical reaction, high costs, and stability under the normal operating conditions of the biosensor [104,105]. Such problems associated with enzyme-based sensors have steered people to develop non-enzyme glucose biosensors, which allow glucose to be oxidized directly on the electrode surface [106]. Lee’s lab first reported the production of an MN-based three-electrode integrated as well as non-enzyme electrochemical sensors, followed by the in vitro characterization of such kind of sensor for detecting glucose levels, as shown in Figure 3A. The silicon was later dried and wet etched to generate a 15 × 15 array of tall (around 380 µm) sharp silicon MN. The iron catalyst was deposited using a SU-8 shadow mask to make the working and counter electrode. A multi-walled carbon nanotubes (MWCNTs) forest was grown on the silicon MN array as well as platinum nanoparticles were electrodeposited for fabricated a working electrode. The silver was deposited on the Si MN array using a shadow mask as well as chlorinated to make the Ag/AgCl, which acts as a reference electrode. The prepared glucose biosensor combines an MN array for potential trans-dermal extraction of body fluid as well as a Platinum-based nanoparticle embedded with a CNT array, which can enhance the electroactive surface area for non-enzymatic electrochemical in vitro detection of the level of glucose. This newly designed MN-based glucose sensor has a potential application for the treatment of painless diabetes. [96]. The Park team generated a patch kind of enzyme that is free of the biosensor with a 3D MN array and Pt black sensing layer, which can be used for minimally invasive as well as applications in continuous monitoring of the level of glucose, as shown in Figure 3B. The 3D MN are manufactured using a technique called micro-fabrication as well as 316L medical-grade stainless steel was used to minimalize the pain of insertion into the skin. The proposed MN array contains a Pt black working electrode and an Ag/AgCl reference electrode, which were formed by a technique called electroplating. The glucose sensor showed a correlation coefficient (0.961) in the range of 0 to 36 mM (648 mg/dL) under the same potential of 400 mV in a two-electrode system. An exciting in vivo experiment exhibited that a sensor can be inserted into the back of the rabbit and can be used to detect the glucose level change in the subcutaneous [97]. Cho et al. reported a novel MN-based amperometric non-enzymatic glucose sensor for painless and continuous monitoring of glucose. The sharp stainless-steel MN (3 × 5) was first coated with a thin Au layer, then Nf and platinum black were sequentially coated onto the tip of gold-coated MN and used for non-enzymatic (direct) sensing of glucose. At a working potential of +0.12 V vs. Ag/AgCl, the sensor shows a linear range from 1 to 40 mM at near neutral pH values, a sensitivity as high as 175 μA mM^−1^ cm^−2^ [98]. A few years later, Cho investigated their more optimized non-enzymatic sensor to determine glucose levels via a minimally invasive procedure in ISF and animal models. The sensitivity of the sensor in PBS and ISF are 5.786 ± 0.17 μA mM^−1^ cm^−2^ and 4.380 ± 0.21 μA mM^−1^ cm^−2^, respectively. The animal in vivo experiment showed this highly porous platinum black (Pt-black)-modified MN electrode arrays have a good correlation between the ISF and capillary glucose levels. This device achieved continuous monitoring of glucose for 7 days [99]. 

#### 3.2.3. Wearable MN-Based Glucose Sensor

**Figure 4:**
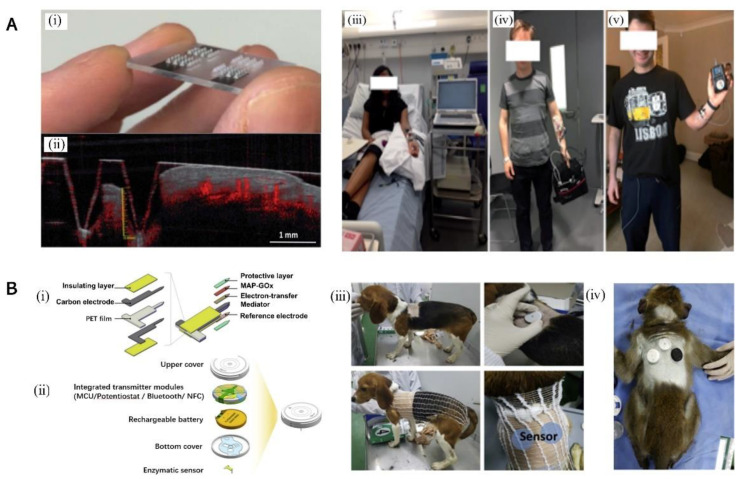
(**A**) (i) Figure showing the designed MN arrays which can be used to obtain optical coherence tomography (OCT) images; (ii) an OCT image was used to investigate at the axial displacement of the MN. Moreover, figure shows the instrumentation used in different stages of the clinical study. (iii) CHI potentiostat on a trolley; (iv) Emstat potentiostat; (v) potentiostat on a printed circuit board, “reprinted with permission from Ref. [100]. 2018, the Royal Society of Chemistry”; (**B**) (i) The PET film used as substrate in this work, and the carbon which is coated on its surface, and it is covered with an insulating layer. The sensor materials contain a protective layer (PPS), an electron-transfer mediator (FA), immobilized GOx with MAP, and silver/silver chloride as reference electrode. (ii) A transmitter, has on/off button on upper side, integrated the transmitter modules, the lithium-ion polymer battery, and the MN located at the bottom cover. The diameter and height of the assembled transmitter was 33 mm and 7.5 mm respectively. (iii) Illustration of inserting procedures of the sensors on a beagle for testing the algorithm used in temperature compensation, (iv) Diagram showing the inserting of sensors on the diabetic cynomolgus of monkey for testing compensation algorithm of the time-lag, “reprinted with permission from Ref. [101]. 2019, Elsevier”.

Wearable sensor technology has advanced rapidly in recent years to provide tremendous opportunities for improving personalized healthcare [20,107]; up to now, several wearable electrochemical sensors have been developed to monitor glucose under human subcutaneous ISF. Sharma et al. reported a solid-MN-array-based minimally invasive continuous glucose monitoring system as shown in Figure 4A. The MN array was fabricated and produced by casting the structures in epoxy-based negative photoresist material (SU8 50), crosslinking, and then metalizing them with Pt or Ag to obtain the working and reference electrodes, respectively [108,109]. The glucose biosensors were functionalized with a GOx enzyme film using an electro-polymerization method in polyphenol solution. After the MN was inserted into the forearm for 24 h, no inflammation or skin irritation was reported by the volunteers. These amperometric sensors can yield currents that can monitor the level of glucose concertation, showing a clinically acceptable correlation. The analysis in people with type 1 diabetes showed a mean absolute relative difference (MARD) of around 9% (with respect to the glucose level in vein), with more than 94% of the data points in the zones of A and B associated with the Clarke error grid [100]. Cho and Cha et al. gave a novel enzymatic continuous glucose monitoring system with a mussel adhesive protein (MAP) employing an enzymatic glucose sensor to enhance the long-term stability as shown in Figure 4B. Their glucose sensor devices include a transmitter, potentiostat, analog-to-digital converter, and Bluetooth system, which can thus provide a hypoglycemia alert to the diabetic patient. The pre-clinical study in diabetic beagles showed the novel glucose sensor has a be a better performance than the commercial continuous glucose monitoring system over the whole concentration range (70–400 mg dL^−1^). Then, the proposed glucose sensor was used on human volunteers by the oral glucose tolerance test (OGTT) and a meal tolerance test (MTT) protocol. The pilot-clinical trials demonstrated that the proposed CGMS successfully measured the glucose levels in ISF and maintained a high accuracy with a mean absolute relative difference (MARD) of 7.49%; 99.7% of all measurements fell in the Clarke error grid A and B zones [101].

To address the practicality issues of epidermal ISF -measuring, Mercier and Wang et al. reported a miniaturized, fully integrated, wirelessly operation-based MN technology which was used to monitor ISF biomarkers continuously on human participants who are behaving freely. The MN microelectrodes were manufactured from a poly (methyl methacrylate) (PMMA) stock material using a microcomputer numerical control (CNC) machining of the MN microelectrodes. The working, counter, and reference electrodes are obtained by sputtered metal (Cr/Pt/Ag) and followed by etching with Ag, then again chloritization of the Ag to Ag-AgCl as the reference. Then, they prepare two integrated circuits, which include an electrochemical analog front end (AFE) as well as a Bluetooth low-energy (BLE) system-in-package (SiP). The developed MN biosensors exhibited excellent potential in finding the three target analytes from ISF within wide dynamic ranges. The dynamic ranges were found to be 0–40 mM for glucose, 0–100 mM for alcohol, and 0–28 mM for lactate, with limits of detection of 0.32, 0.15, and 0.50 mM, respectively, as calculated by signal to noise ratio of 3. The on-body performance of all five participants based on the MN array biosensor was determined for glucose sensing with a mean absolute relative difference (MARD) of 8.83% (95 paired datapoints). More importantly, the sensor achieved personalized responses from 220 to 304 mg h dL^−1^. Furthermore, the validation of this technology in large populations with concurrent sensor readouts via centralized laboratory tests determined the robustness and utility of real-time monitoring of several biomarkers in ISF [103].

#### 3.2.4. Raman-MN Glucose Sensors

The optical techniques have been widely explored for intradermal measurements of important biomarkers from skin non-invasively, such as confocal laser scanning microscopy and autofluorescence spectroscopy technologies. However, the fluorescence-based tool suffers from the over-lapping fluorescence signal of the intrinsic fluorophore molecules, which hinders the in vivo detection capability of this technique. Among those optical techniques, Raman spectroscopy has been paid more attention to as it provides structural and morphological information. However, the application of Raman spectroscopy as an in vivo intradermal analytical tool has been so far limited because the Raman signal of the endogenous biomolecules is weak [15]. 

The surface-enhanced Raman spectroscopy (SERS) is used to amplify the Raman signals up to 10^14^ folds using metallic surfaces [110]. In 1977, Van Duyne’s group [111] and Creighton’s lab [112] independently showed the presence of noble metal substrates such as gold as well as silver with nanostructured features at the origin of the dramatic signal enhancement. After which, research has been carried out to understand and characterize the mechanism of this kind of phenomenon [113,114]. There are two underlying principles that can be used to explain SERS; one is electromagnetic (EM) [115] field enhancement via the localization of optical fields in metallic nanostructures, and another one is a chemical enhancement (CE) [116], which is from a cross-section of the molecule in contact with metal nanostructures [117,118]. The EM enhancement, as well as CE, require a very small distance between the analyte molecule and the metallic surface, and the amplification enhancement factor (EF) of SERS is on the order of 10^8^–10^14^. The SERS has been widely used these days as the best analytical tool in trace amounts [119,120]. 

Initially, Clement Yuen and Quan Liu proposed for the first time that MN can be used as a tool to solve the limitation of Raman transmission depth in the skin. They coated thick Ag films onto steel microneedles in MN patches (AdminPatch 200, AdminMed, Sunnyvale, CA, USA) based SERS probe for sensitive detection of glucoseat a depth of more than 700 μm in simulated skin [27]. This work showed the potential of using MN for simple in vivo intradermal SERS measurements of glucose with clinical relevance. However, the wide use of these stainless steel causes a strong skin immune response and would yield potential reuse of MN without adequate sterilization. The Liu group’s subsequent research has come up with an effective solution. They first designed a hollow agarose MN coated with a silver layer for SERS detection of glucose molecules [28]. The bio-friendly polymers agarose materials exhibited more biocompatible than stainless steel even if the MN was broken inside the skin. More importantly, the Ag-coated agarose MN quantifies glucose inside the skin phantoms with a wide range from 5 to 150 mM within 10 s. Unfortunately, Ag-coated agarose MN research for direct intradermal measurements has the following issues: firstly, there is no in vivo efficacy demonstration, which can simply cause permanent skin damage, and secondly, the single needle structure makes it difficult to maintain stability in the simulated skin. To promote the application of Raman-MN technology in vivo measurement of glucose, Ju and Liu developed a novel sensor that is based on a polymethyl methacrylate MN(PMMA MN) array for direct in situ intradermal measurements [121]. The thin silver film is wrapped on the PMMA MN for the enhancement of Raman signals. After being modified with the glucose capture agent of 1-decanethiol (1-DT), the functional MN array was demonstrated for the first time to achieve in vivo intradermal measurements of glucose from ISF in a type I diabetes mouse model. This new Raman-microneedle has the below-mentioned benefits in comparison with the previously mentioned MNs, which are used for intradermal measurements: (1) the PMMA MN array has enough mechanical strength to puncture the mice skin, which can keep the morphology of the needle, while dwelling in the skin. (2) The high light transmission of PMMA enables laser light as well as Raman signals to transmit through the MN, which subverted the traditional idea and reserve the optical transmission channel in the MN (3) The MN array shows the potential biological application, where the visible evidence is the imprints created by the MN array and it can recover well within 10 min without any side effects. 

In brief, the Raman-MN technology passed its last hurdle for the in vivo detection of glucose; with further improvement and proper validation, this polymeric MN array-based SERS biosensor has the potential to develop a new painless wearable glucose detection instrument in the future.

## 4. Conclusions, Prospects, and Challenges

In this review, we have presented the contributions of the MN platform for the minimally invasive monitoring of glucose in the ISF. The ISF is known to be a similar glucose concentration in comparison with the blood, and it is considered a potential body fluid sample to supplant the current blood-based standard. Great progress has been made in recent years regarding the synthesis, characterization, and application of the MN; despite the many challenges faced, more and more bio-friendly materials have been adopted to design different shapes of the MN according to the clinical requirements. However, there is still much work to be performed in terms of attaining precisely controlled and reproducible prepared MN with control over their size, shape, composition, and structure, and this requires the fields of chemistry, physics, electronics, and materials science to make the earnest unremitting endeavor. 

Recently, MN-based glucose sensors have seen tremendous progress, researchers, and people with expectations for this type of technology increasingly believe that MN devices can achieve non-invasive and continuous monitoring of glucose in diabetes management, and it is considered the most scientific method so far. In this review, we focus on the new progress of MN glucose sensor for ISF extraction and measurement of glucose after MN coupled with colorimetry, electrochemical, and Raman spectroscopy analytical technology. We were able to realize that functional MN is undoubtedly the most central part of the above MN-based glucose sensor, which makes it more active in the developing wearable biosensor field. Currently, the electrochemical-based MN glucose biosensors dominate the most wearable glucose biosensor; with the integration of electrochemical systems, self-powered, and wireless electronics, the measurement of data was read from an accompanying smartphone app for visualization. The above research aspects meet the need for the development of wearable glucose biosensors, which are intelligent, miniaturized, wearable, and data visualization. The optical-based MN glucose sensors are another important wearable technology, which can overcome the shortcomings of electrochemical, owing to their self-power-free, non-invasive nature, and there is no need to integrate a signal transmission system. Raman technology has gradually solved the key scientific and technical problem of weak Raman signal after the laser transmission MN, which exhibited a huge potential in the development of painless glucose monitoring devices. It is worth pointing out that the functional MN is the key device of this kind of sensor, which is more convenient to design and prepare devices worn on the human skin.

In future research, as a high-frequency transdermal sensor, it will be an inevitable trend to integrate MN made from bio-friendly materials, which have the potential for long-term retention and wearing. More importantly, the MN design needs to be considered for variation in gender, the body mass index (BMI), and ethnicity of the wearer. It can be predicted that the product will dominate the wearable glucose sensors market that is based on electrochemical technology soon. Future more, the developments of the system will be focused on several aspects: Enzyme stability of enzyme-based electrode, biofouling issues of an enzyme-free electrode, extension of test time, calibration of sensor data in real-time, power supply, measurement of data of real-time analysis issues, etc., [103].

The optical technology, especially Raman spectroscopy, has the potential to prepare wearable glucose sensors. Indeed, some researchers have already made a major contribution to the development of Raman-based MN glucose sensors; currently, the miniaturization of the Raman spectrum is the final breakthrough point for such sensors. Take an optimistic view, MN-based wearable glucose sensor empowered multiplexed sensing devices to be available in the foreseeable future.

## Figures and Tables

**Table 1:** Comparison between the different electrochemical-MN performances for the detection of glucose.

MN Description	Type Sensor	Analytical Technique	Analytical Parameters	Application	Ref.
Solid, Hollow/PPD/GOx	Enzyme EC	Amperometric	Linear range: 0–14 mM LOD: 0.1 mM	None	[83]
Solid, Au/GOx	Enzyme EC	Amperometric	Linear range: 0–25 mM LOD: 0.1 mM	ISF	[84]
Hollow/carbon paste/GOx/TTF	Enzyme EC	power density	Linear range: 5–25 mM LOD: 0.1 mM	Artificial ISF	[85]
Solid/PEDOT/GOx	Enzyme EC	Amperometric	Linear range: up to 396 mg/dL (dry 7 days)	None	[86]
PVDF-Nf/GOx	Enzyme EC	Amperometric	Linear range: 0–20 mM LOD: 0.1 mM	Mice	[87]
Au/MPA/GOx	Enzyme EC	Cyclic voltammetry	Linear range: 0–400 mg/dL	ISF	[88]
Solid/Au/FcCOOH/GOx	Enzyme EC	Amperometric	Linear range: 2–13.5 mM	None	[89]
Hollow/Pt/GOx	Enzyme EC	Amperometric	up to 500 mg/dL	Volunteer	[90]
Solid/FAD-GDH/FcSH/h-PG/Au	Enzyme EC	Amperometric	Linear range: 0.1–10 mM	Artificial ISF	[91]
AuMN/pTCA-GOx	Enzyme EC	Amperometric	Linear range: 0.05–20 mM	Volunteers	[92]
Solid/Au/OPPy/AuNPs/GOx/Nf	Enzyme EC	Amperometric	Linear range: Up to 2.6 mM LOD: 0.04 mM	None	[93]
Solid/Silk/polyols/GOD	Enzyme EC	Amperometric	Linear range: 1.7–10.4 mM	None	[94]
Solid/Au/GOD	Enzyme EC	Amperometric	Linear range: 3–24 mM LOD:0.048 mM	Mice	[26]
Solid/Au-Si-MNA/Fc-PAMAM/GOx	Enzyme EC	Amperometric	Linear range: 3.6–6.0 mM	Mice	[95]
Solid/CNTs/Pt NPs	Non-Enzyme EC	Amperometric	Linear range: 3–20.0 mM	None	[96]
Solid/Pt black	Non-Enzyme EC	Amperometric	Linear range: up to 36 mM LOD: 0.05 mM	Rabbit	[97]
Solid/Au/Pt black/Nf	Non-Enzyme EC	Amperometric	Linear range: 1–40 mM LOD: 0.023 mM	blood serum	[98]
Solid/MN/Au/Pt black/Nf	Non-Enzyme EC	Amperometric	Linear range: 1–20 mM	Rat	[99]
Solid/Pt	Non-Enzyme EC	Amperometric	MARD = 9%, 96.6% in Zone A and B(CGM)	volunteer	[100]
Solid/MAP/GOx	Enzyme EC	Amperometric	Linear range: 100–400 mg/dL	Beagle volunteer	[101]
Solid/Au/GOx	Enzyme EC	Amperometric	Linear range: 1–40 mM	rat volunteer	[102]
Solid/Pt/PPD/GOx-Chitosan/PVC	Enzyme EC	Amperometric	Linear range: 0–40 mM	volunteer	[103]

Table Abbreviation, poly(o-phenylenediamine) (PPD), Glucose oxidase (GOx), tetrathiafulvalene (TTF), poly(3,4-ethylenedioxythiophene) (PEDOT), Nafion (Nf), Polyvinylidene fluoride (PVDF), 3-Mercaptopropionic acid (MPA), ferrocene monocarboxylic acid (FcCOOH), flavin adenine dinucleotide glucose dehydrogenase (FAD-GDH), terthiophene carboxylic acid (TCA), overoxidized polypyrrole (OPPy), glucose oxidase (GOD), MN array (MNA), ferrocene cored poly(amidoamine), dendrimers (Fc-PAMAM), Multi-walled carbon nanotubes (MWCNTs), mussel adhesive protein (MAP), poly-o-phenylenediamine (PPD), polyvinyl chloride (PVC).

## Data Availability

Not applicable.
